# Decision Tree Methods for Predicting Surface Roughness in Fused Deposition Modeling Parts

**DOI:** 10.3390/ma12162574

**Published:** 2019-08-12

**Authors:** Juan M. Barrios, Pablo E. Romero

**Affiliations:** Department of Mechanical Engineering, University of Cordoba, Medina Azahara Avenue, 5–14071 Cordoba, Spain

**Keywords:** fused deposition modeling (FDM), PETG, surface roughness, data mining, decision tree, C4.5, random forest, random tree

## Abstract

3D printing using fused deposition modeling (FDM) includes a multitude of control parameters. It is difficult to predict a priori what surface finish will be achieved when certain values are set for these parameters. The objective of this work is to compare the models generated by decision tree algorithms (C4.5, random forest, and random tree) and to analyze which makes the best prediction of the surface roughness in polyethylene terephthalate glycol (PETG) parts printed in 3D using the FDM technique. The models have been created using a dataset of 27 instances with the following attributes: layer height, extrusion temperature, print speed, print acceleration, and flow rate. In addition, a dataset has been created to evaluate the models, consisting of 15 additional instances. The models generated by the random tree algorithm achieve the best results for predicting the surface roughness in FDM parts.

## 1. Introduction

Additive manufacturing or 3D printing techniques allow small batches of parts to be produced directly, economically, and flexibly [1]. There are different additive manufacturing techniques; however, fused deposition modeling (FDM) printers are the most extended due to their low cost and the wide variety of materials that can be used [2,3,4].

FDM printers offer a large number of print parameters: print temperature, layer height, print speed, print acceleration, and flow rate, among others. When a part is printed, it is difficult to predict a priori if it will receive an adequate surface finish [5,6]. 

Data mining techniques are used to improve the quality of processes and products based on data gathered from previous experiences [7,8,9]: they are used to find out which parameters are most influential in surface finishing in electrical discharge machining (EDM) processes [10]. They are also used to predict the wear of a tool in milling processes [11] or to increase the accuracy of high-speed machining of titanium alloys [12].

Data mining techniques can be classified into supervised (classification, regression) and unsupervised (clustering, association rules, correlations). Classification techniques are widely utilized [9]. To use these techniques, different classes must be established in which each instance in the database must belong to a class; the rest of the attributes of the instance are used to predict that class. The objective of these algorithms is to maximize the accuracy ratio of the classification of new instances [13].

Decision trees are one of the most widely used classification techniques in data mining [13]. Tree nodes represent a condition relative to a given attribute. The leaves indicate the number of instances that belong to a class and that satisfy the conditions imposed in the previous nodes. By means of this type of algorithms, it is possible to create models that allow for predictions such as whether a register with certain attributes will belong to one class or another. There are several classification algorithms: C4.5 [14], random forest [15], random tree [16].

Previous work has focused on the use of classification techniques in additive manufacturing processes. Wu et al. [17,18] applied random forest, k-nearest neighbor, and anomaly detection techniques to detect defects caused by a cyberattack on an FDM printer during part fabrication. Amini and Chang [19] used classification techniques to reduce defects in metal parts manufacturing processes using selective laser melting (SLM) printers. Recently, Li et al. [20] have generated and checked models using different machine learning algorithms to predict the surface roughness of 3D printed parts using FDM; in this case, the authors used a design of experiments with three variables (layer height, print temperature, and print speed/flow rate) and measured the roughness in a unique direction. 

The objective of this work is to analyze which decision tree algorithm (C4.5, random forest, or random tree) is the best to predict the surface finish (*R*_*a*,0_ and *R*_*a*,90_) of a FDM printed part. Data mining models have been developed from a training dataset consisting of 27 instances with attributes of layer height, print temperature, flow rate, print speed, print acceleration, *R*_*a*,0_ class, and *R*_*a*,90_ class. These models have been tested using a dataset with 15 additional instances.

## 2. Materials and Methods

**Figure 1:**
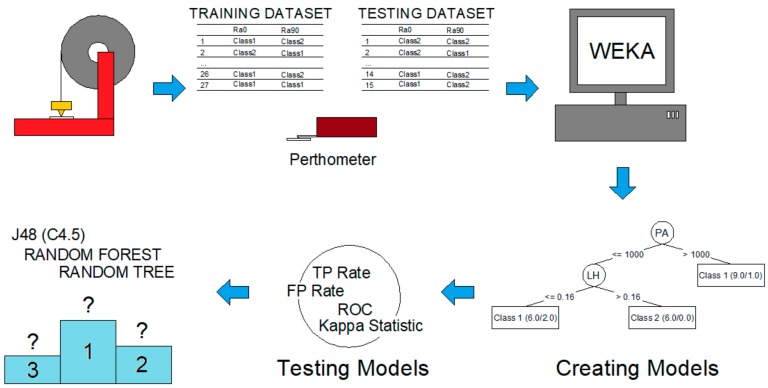
Different stages that compose the methodology followed in the present work: 3D printing, surface roughness measurements, data mining, models generation, models testing, and comparison between algorithms.

Figure 1 shows the different stages that constitute the methodology followed in this work: 3D printing, surface roughness measurements, data mining, models generation, models testing, and comparison between algorithms.

### 2.1. 3D Printing and Surface Roughness Measurements

To carry out the present study, 27 specimens of dimensions 25.0 mm × 25.0 mm × 2.4 mm were printed, following a design of fractionated orthogonal experiments, with five factors and three levels. The parameters used and the values assigned to each level are shown in Table 1.

The specimens were designed using SolidWorks software. The selection of values for print parameters and numerical code (NC) generation was done using CURA software. The specimens were manufactured using an Ender 3 printer, with a diameter nozzle equal to 0.4 mm. 

The polyethylene terephthalate glycol (PETG) filament was supplied by Smart Materials 3D (Smart Materials 3D, Alcalá la Real, Spain). Although there are not many published works on PETG, it is a filament increasingly used in the industry, having mechanical characteristics similar to ABS but printed with the ease of a PLA [21].

**Figure 2:**
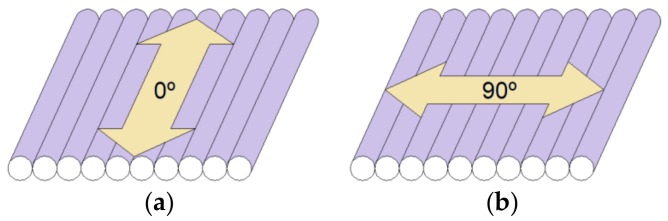
Measurement of surface roughness in the direction parallel to the direction of extrusion (**a**) and in the direction perpendicular to the direction of extrusion (**b**).

Once printed, the roughness of the specimens was measured using a Mitutoyo perthometer model SJ-201 (Mitutoyo, Kawasaki, Japan). Five measurements were made in each specimen and direction, 0° and 90°, as seen in Figure 2. The representative value was calculated as the arithmetic mean of the five measurements. The resulting values are shown in Table 2.

The models were generated using the free software WEKA (Waikato Environment for Knowledge Analysis). The roughness values were divided into two classes (class 1 and class 2) from the mean of the values of *R*_*a*,0_ and *R*_*a*,90_. For *R*_*a*,0_, class 1 includes 0–4.43 μm and class 2 is from 4.43 to 10.64 μm. For *R*_*a*,90_, class 1 includes 0–11.63 μm and class 2 includes 11.63–32.99 μm. To validate the model obtained, 15 additional specimens were printed using random values for the parameters under study, as seen in Table 3. Likewise, five roughness measurements were performed on each specimen and direction, obtaining the representative values by means of the arithmetic mean. 

### 2.2. Data Mining and Decision Trees

The data mining process consists of several steps: (1) integration and data collection, which coincides with the previous paragraph; (2) selection, cleaning and transformation, in which the data were prepared in .arff format, which are files used by WEKA; (3) data mining, consisting of applying the algorithms to the data and generating patterns and models; (4) evaluation and interpretation of the information generated; (5) generation of knowledge and decision making.

In this work, three algorithms based on decision trees are compared: J48 (a variation of WEKA for C4.5), random forest, and random tree. The algorithms are briefly presented below.

#### 2.2.1. J48 (C4.5)

The J48 is WEKA’s version of the C4.5 algorithm. Algorithm C4.5, created by Quinlan [14], allows the generation of decision trees. It is an iterative algorithm that consists of dividing the data in each stage into two groups using the concept of information entropy. This partitioning process is recursive and stops when all records of a sheet belong to the same class or category.

#### 2.2.2. Random Forest

The random forest method was developed by Breiman [15]. It consists of constructing multiple decision trees using random combinations and orders of variables before constructing a random tree using bootstrap aggregating (also known as bagging). This highly accurate algorithm is capable of handling hundreds of variables without excluding any [16].

#### 2.2.3. Random Tree

The random tree is an algorithm halfway between a simple decision tree and a random forest. Random trees are a set of predictor trees called forest. The classification mechanisms are as follows: the random tree classifier obtains the input characteristic vector, classifies it with each tree in the forest, and produces the class label that received the most “votes” [13].

## 3. Results

This work proposes the use of decision trees and data mining techniques to predict which values should be selected for the print parameters of PETG flat specimens, manufactured by FDM. The dataset used as a training dataset to develop the models is shown in Table 4.

**Figure 3:**
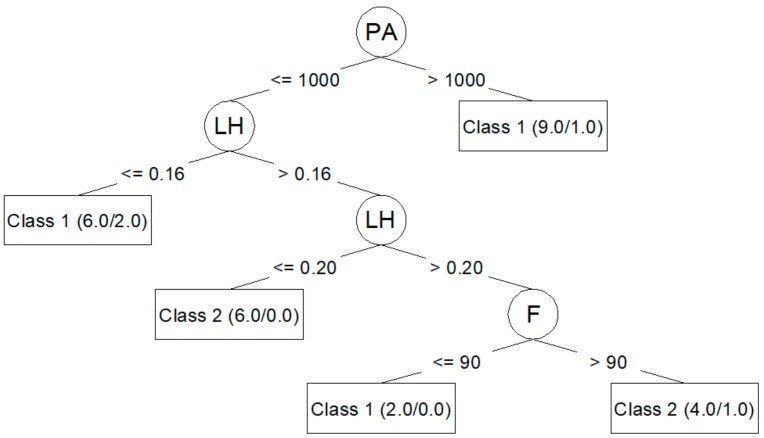
J48 (C4.5) decision tree for *R*_*a*,0_.

**Figure 4:**
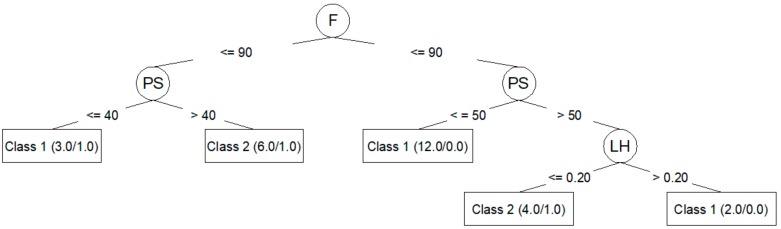
J48 (C4.5) decision tree for *R*_*a*,90_.

Algorithm J48 (C4.5) generates decision trees that can be represented graphically. Figure 3 shows the decision tree generated for *R*_*a*,0_ from the corresponding training dataset. Figure 4 shows the decision tree generated for *R*_*a*,90_.

Once the models have been generated using the algorithms studied, they have been checked using the dataset shown in Table 5. The results of each algorithm for the prediction of *R*_*a*,0_ are shown in Table 6 and Table 7. Likewise, the results for the prediction of *R*_*a*,90_ are presented in Table 8 and Table 9. Table 10 shows the time used by each algorithm to construct and validate the models. 

The J48 algorithm allows for the generation of simple trees that can be easily understood and interpreted, as seen in Figure 3 and Figure 4. In these trees, it is clear which parameters are most important to reduce *R*_*a*,0_ (PA, LH, F) and *R*_*a*,90_ (F, PS, LH). However, the models created by means of the J48 algorithm are those that adjust the worst to the test data, as seen in Table 6 and Table 8: a 60.00% success rate for *R*_*a*,0_; a 73.33% success rate for *R*_*a*,90_; and a negative kappa statistic in both cases. Table 7 and Table 9 show that the precision for the models created using the J48 algorithm is lower than that achieved by the rest of the models, and that the value of the area under the ROC is very low.

The random forest algorithm slightly improves the results of J48, as seen in Table 6 and Table 8; the model created using this algorithm provides 66.67% success for *R*_*a*,0_ and 80.00% success for *R*_*a*,90_. In this case, the kappa statistic is close to zero for *R*_*a*,0_ and negative for *R*_*a*,90_. Table 7 and Table 9 show that the precision of both models increases, as does the area under ROC. This algorithm does not generate trees that can be plotted.

The models generated by the random tree algorithm are the ones that obtain the best results in this case, as seen in Table 6 and Table 8, with an 80% success rate for *R*_*a*,0_ and 86.67% success rate for *R*_*a*,90_. It obtains a positive kappa statistic in both cases: 0.2857 for *R*_*a*,0_ and 0.5946 for *R*_*a*,90_. These values can be classified as fair and moderate according to Table 11. Table 7 and Table 9 show that the precision of both models is higher than in the cases analyzed above; the area under ROC is 0.673 for the *R*_*a*,0_ model and 0.923 for the *R*_*a*,90_ model. In addition, as can be seen in Table 10, this algorithm took the least time to build the models.

## 4. Discussion

In the present work, three classification tree algorithms were used to predict the surface finish of FDM-printed PETG flat specimens. The algorithms used were J48, random forest, and random tree. The software used to generate the models was WEKA [13].

The J48 algorithm has been widely used to study problems in the manufacturing area related to quality improvement in production processes [10,22]. In this work, the J48 generated models that classified training dataset data very well. Additionally, this algorithm made it possible to create trees that can be plotted and that are easily understood [10,16,23,24]. This algorithm also allowed for the identification of the most influential parameters in roughness: PA, LH, and F for *R*_*a*,0_; F, PS, and LH for *R*_*a*,90_. These results coincide with those obtained by the authors in previous works [25]: PA is the parameter with the greatest influence on *R*_*a*,0_; F, PS, and LH are the parameters with the greatest influence on *R*_*a*,90_. However, in this case, the models created with J48 algorithm were not able to predict the data used in the test. This may be related to overfitting problems [13,26].

In the problem addressed, the random forest algorithm obtained better results than J48, as could be expected from the literature [27,28,29]. Although it does not generate a graphical model, this algorithm is widely used by other authors in problems similar to the one studied [30]. 

In this work, the random tree algorithm generated the best models for *R*_*a*,0_ and *R*_*a*,90_. Previously, other authors have also chosen this algorithm for its properties [31]. An additional advantage of this algorithm is its calculation time, which is the fastest of those studied [32].

## 5. Conclusions

In the present work, three models were created and compared to predict the surface roughness of flat pieces printed on PETG by FDM. For this purpose, data mining classification were used, such as J48 (C4.5), random forest, and random tree. The software used to generate the models was the open source software WEKA.

The model generated by random tree obtains better results. It correctly classifies a priori 80% of the instances in the case of *R*_*a*,0_ and 86.67% of the instances in the case of *R*_*a*,90_. It is the only algorithm of the three evaluated that achieves a positive kappa statistic, qualified as fair for *R*_*a*,0_ and moderate for *R*_*a*,90_. It obtains the highest accuracy and area under ROC and is also the fastest algorithm of the three analyzed.

In future works, we intend to study whether the decision trees can be used to generate models that allow for the prediction of a better dimensional accuracy of the parts manufactured by FDM. The impact of other print factors on the surface properties of printed parts, such as nozzle diameter, will also be studied.

## Figures and Tables

**Table 1:** Factors and levels used in design of the experiments (DOE).

Print Parameter	Level 1	Level 2	Level 3
Layer height (LH), mm	0.16	0.20	0.24
Temperature (T), °C	240	245	250
Print speed (PS), mm/s	40	50	60
Print acceleration (PA), mm/s^2^	500	1000	1500
Flow rate (F), %	90	100	110

**Table 2:** Training dataset: design of experiment (*L*_27_), according to Taguchi method: layer height (LH), print temperature (T), print speed (PS), print acceleration (PA), and flow rate (F).

No.	LH (mm)	T (°C)	PS (mm/s)	PA (mm/s^2^)	F (%)
1	0.16	240	40	500	90
2	0.16	240	40	500	100
3	0.16	240	40	500	110
4	0.16	245	50	1000	110
5	0.16	245	50	1000	90
6	0.16	245	50	1000	100
7	0.16	250	60	1500	100
8	0.16	250	60	1500	110
9	0.16	250	60	1500	90
10	0.20	240	50	1500	100
11	0.20	240	50	1500	110
12	0.20	240	50	1500	90
13	0.20	245	60	500	90
14	0.20	245	60	500	100
15	0.20	245	60	500	110
16	0.20	250	40	1000	110
17	0.20	250	40	1000	90
18	0.20	250	40	1000	100
19	0.24	240	60	1000	110
20	0.24	240	60	1000	90
21	0.24	240	60	1000	100
22	0.24	245	40	1500	100
23	0.24	245	40	1500	110
24	0.24	245	40	1500	90
25	0.24	250	50	500	90
26	0.24	250	50	500	100
27	0.24	250	50	500	110

**Table 3:** Test dataset: design of experiment (*L_27_*), according to Taguchi method: layer height (LH), print temperature (T), print speed (PS), print acceleration (PA), and flow rate (F).

No.	LH (mm)	T (°C)	PS (mm/s)	PA (mm/s^2^)	F (%)
1	0.14	240	15	200	95
2	0.14	236	18	300	105
3	0.18	238	15	300	115
4	0.18	243	20	200	95
5	0.14	246	35	400	105
6	0.23	248	46	400	95
7	0.24	243	45	600	103
8	0.30	230	56	2000	100
9	0.30	250	60	1600	110
10	0.20	251	70	1500	102
11	0.20	249	85	1200	100
12	0.28	249	100	1200	98
13	0.28	237	25	1100	90
14	0.14	238	21	800	100
15	0.10	239	50	600	110

**Table 4:** Training dataset: results for surface roughness (*R*_*a*,0_, *R*_*a*,90_).

Test	*R*_*a*,0_ (µm)	*R*_*a*,0_ Class	*R*_*a*,90_ (µm)	*R*_*a*,90_ Class
1	10.648	Class2	12.240	Class2
2	0.916	Class1	6.464	Class1
3	1.126	Class1	9.160	Class1
4	2.428	Class1	32.994	Class2
5	1.800	Class1	5.504	Class1
6	8.814	Class2	10.922	Class1
7	4.552	Class2	23.650	Class2
8	1.370	Class1	14.458	Class2
9	0.954	Class1	5.414	Class1
10	1.462	Class1	23.470	Class2
11	1.666	Class1	9.050	Class1
12	1.554	Class1	10.074	Class1
13	6.258	Class2	20.088	Class2
14	7.788	Class2	15.368	Class2
15	10.172	Class2	12.560	Class2
16	9.744	Class2	10.186	Class2
17	4.696	Class2	5.462	Class1
18	5.112	Class2	5.330	Class1
19	4.274	Class2	10.668	Class1
20	6.994	Class1	8.214	Class1
21	5.868	Class2	6.056	Class1
22	3.796	Class2	8.680	Class1
23	3.054	Class1	5.720	Class1
24	3.702	Class1	6.804	Class1
25	4.124	Class1	19.654	Class1
26	4.682	Class1	8.964	Class2
27	2.256	Class2	7.122	Class1

**Table 5:** Testing dataset: results for surface roughness (*R*_*a*,0_, *R*_*a*,90_).

Test	*R*_*a*,0_ (µm)	*R*_*a*,0_ Class	*R*_*a*,90_ (µm)	*R*_*a*,90_ Class
1	1.026	Class1	4.462	Class1
2	1.178	Class1	2.656	Class1
3	2.064	Class1	3.97	Class1
4	1.126	Class1	7.192	Class1
5	1.984	Class1	9.24	Class1
6	1.252	Class1	8.276	Class1
7	1.026	Class1	4.462	Class1
8	1.744	Class1	7.732	Class1
9	5.906	Class2	11.408	Class1
10	3.99	Class1	12.082	Class2
11	1.182	Class1	6.392	Class1
12	1.008	Class1	6.622	Class1
13	6.466	Class2	14.002	Class2
14	0.606	Class1	6.828	Class1
15	1.846	Class1	4.758	Class1

**Table 6:** Indicators to compare the models generated by the studied algorithms to predict *R*_*a*,0_.

Indicator	J48	Random Forest	Random Tree
Correctly Classified Instances	60.00%	66.67%	80.00%
Incorrectly Classified Instances	40.00%	33.33%	20.00%
Kappa statistic	−0.2162	0.1176	0.2857
Mean absolute error	0.4926	0.429	0.2
Root mean squared error	0.595	0.474	0.4472
Relative absolute error	106.60%	92.84%	43.28%
Root relative squared error	128.39%	102.29%	96.50%

**Table 7:** Detailed precision parameters achieved by each algorithm for the *R*_*a*,0_ prediction model.

Detailed Accuracy (weighted av.)	J48	Random Forest	Random Tree
True Positive (TP) Rate	0.600	0.667	0.800
False Positive (FP) Rate	0.908	0.474	0.454
Precision	0.709	0.807	0.839
Recall	0.600	0.667	0.800
F-measure	0.650	0.716	0.816
MCC	−0.237	0.139	0.294
ROC Area	0.154	0.692	0.673
PRC Area	0.697	0.868	0.819

**Table 8:** Indicators to compare the models generated by the studied algorithms to predict *R*_*a*,90_.

Indicator	J48	Random Forest	Random Tree
Correctly Classified Instances	73.33%	80.00%	86.67%
Incorrectly Classified Instances	26.67%	20.00%	13.33%
Kappa statistic	−0.1538	−0.0976	0.5946
Mean absolute error	0.2556	0.2888	0.1333
Root mean squared error	0.4645	0.3854	0.3651
Relative absolute error	66.17%	74.57%	34.52%
Root relative squared error	116.01%	96.26%	91.20%

**Table 9:** Detailed precision parameters achieved by each algorithm for the *R*_*a*,90_ prediction model.

Detailed Accuracy (weighted av.)	J48	Random Forest	Random Tree
True Positive (TP) Rate	0.733	0.800	0.867
False Positive (FP) Rate	0.887	0.877	0.021
Precision	0.733	0.743	0.933
Recall	0.733	0.800	0.867
F-measure	0.733	0.770	0.883
MCC	−0.154	−0.105	0.650
ROC Area	0.385	0.481	0.923
PRC Area	0.745	0.797	0.916

**Table 10:** Time used by each algorithm to build and validate the model.

Algorithm	Computing Time for *R*_*a*,0_ Model (s)	Computing Time for *R*_*a*,90_ Model (s)
J48	0.11	0.19
Random Forest	0.05	0.34
Random Tree	0.01	0.01

**Table 11:** Strength of concordance for kappa statistic.

Kappa Statistic	Strength of Concordance
0.00	Poor
0.01–0.20	Slight
0.21–0.40	Fair
0.41–0.60	Moderate
0.61–0.80	Substancial
0.81–1.00	Almost perfect
